# Self-reported ongoing adherence to diet is associated with lower depression, fatigue, and disability, in people with multiple sclerosis

**DOI:** 10.3389/fnut.2023.979380

**Published:** 2023-03-01

**Authors:** Maggie Yu, George Jelinek, Steve Simpson-Yap, Sandra Neate, Nupur Nag

**Affiliations:** ^1^Neuroepidemiology Unit, Centre for Epidemiology and Biostatistics, Melbourne School of Population and Global Health, The University of Melbourne, Melbourne, VIC, Australia; ^2^Menzies Institute for Medical Research, University of Tasmania, Hobart, TAS, Australia; ^3^Clinical Research Outcomes Unit, The Royal Melbourne Hospital, Melbourne, VIC, Australia

**Keywords:** multiple sclerosis, health outcomes, fatigue, depression, disability, prospective observational study, diet adherence

## Abstract

**Introduction:**

Increasingly, dietary improvements have been shown to have positive associations with health outcomes in people with multiple sclerosis (pwMS). However, adhering to a MS-specific or high-quality diet may be a challenge. We therefore assessed the level of diet-adherence necessary to improve health outcomes of depression, fatigue, and disability.

**Methods:**

Data from an international population of pwMS followed over 7.5 years (*n* = 671) were analyzed. Self-reported diet quality *via* diet habits questionnaire (DHQ), and adherence to six MS-diets [Ashton Embry Best Bet, McDougall, Overcoming MS (OMS), Paleolithic (Paleo), Swank, and Wahls] were queried at two timepoints. Four levels of diet adherence were assessed: non-adherence at either timepoint; ceased at second timepoint; commenced at second timepoint; and ongoing at both timepoints. Associations between adherence to OMS and high-quality diet (DHQ score > median) with depression, fatigue, and disability, were assessed by log-binomial regression models adjusted for confounders.

**Results:**

Forty-two percent of pwMS reported ongoing-adherence to a MS-diet at both timepoints, OMS (33%), Swank (4%), Wahls (1.5%), other (<1%). Of these, only OMS-diet adherence was analyzed for associations due to data availability. Ongoing-adherence to the OMS-diet or a high-quality diet, was associated with lower depression compared to non-adherence [OMS: Risk ratios (RR) = 0.80, *p* = 0.021; DHQ: RR = 0.78, *p* = 0.009] and ceased-adherence (OMS: RR = 0.70, *p* = 0.008; DHQ: RR = 0.70, *p* = 0.010), respectively. Ongoing-adherence to OMS-diet was associated with lower fatigue (RR = 0.71, *p* = 0.031) and lower severe disability (RR = 0.43, *p* = 0.033) compared to ceased-adherence.

**Conclusion:**

Results suggest potential benefits of adherence to the OMS- or a high-quality diet on MS health outcomes, with ongoing-adherence likely best. Diet modification and maintenance may serve as a point of intervention to manage MS symptoms, especially depression, in pwMS.

## 1. Introduction

Dietary modification is increasingly emerging as a safe and feasible approach to manage symptoms and improve health and wellbeing of people with multiple sclerosis (pwMS) (1). The earliest reports suggesting a role for diet in MS were from epidemiological studies showing that populations living in coastal parts of Norway with more seafood intake had lower frequencies of MS compared to inland populations with diets high in saturated animal fats (2, 3). Subsequent studies based on these observations, showed pwMS adhering to a diet of < 20 g saturated fat per day, had less disability and less mortality over multiple decades’ follow-up (4). These studies lead to the development of the Swank diet in 1950s, which recommends minimized intake of saturated fats and processed food (5).

In addition to the Swank-diet, MS-diets proposed include Overcoming MS (OMS), Ashton Embry Best Bet, McDougall, Paleolithic (Paleo), and Wahls (6, 7). These diets are similar in recommend intake of fruit and vegetables and limited intake of processed foods; and differ in elimination of certain foods. Some studies have shown adherence to MS-diet is associated with improved health outcomes in pwMS (8, 9). For example, the OMS diet, a plant-based whole food (food that has been processed or refined as little as possible), low-saturated fat diet with seafood (10), has been cross-sectionally associated with lower fatigue, depression, and disability (11). Adherence to the OMS diet, as part of a multimodal lifestyle program, was prospectively associated with improved quality of life (QoL), and reduced fatigue and depression among 274 pwMS (12). Adherence to the low-fat, plant-based McDougall resulted in reduced body mass index (BMI) and improved fatigue in a 12-month clinical trial of 61 pwMS (13). Adherence to the Paleo diet, which limits foods that became common when farming emerged, was associated with reduced fatigue and improved QoL in a 3-month clinical trial among 17 pwMS (14, 15). The Wahls diet, a modified Paleo diet to increase intake of nutrients key to neuronal health and limit lectins, has also been shown to reduce fatigue and improve QoL in a clinical trial of 77 pwMS (16). The Ashton Embry Best Bet diet excludes dairy, gluten, legumes, and refined sugars (17); however, associations between this diet with MS health outcomes have not yet been reported. In addition to MS-specific diet programs, many pwMS adhere to the Mediterranean and other anti-inflammatory diets (6, 9) and reported better MS outcomes such as lower fatigue and disability in observational (18, 19) and clinical trial studies (20, 21).

High-quality diets, which emphasize an overall pattern of intake that is high in fruits, vegetables, whole grains, and fish, and low in refined sugars, processed meat, and saturated fat, have been associated with better health outcomes in pwMS (1). Diet quality has been assessed using different measurements such as food diaries, food frequency questionnaire (22), dietary screener questionnaire (DSQ) (23), and the diet habits questionnaire (DHQ). Cross-sectionally, studies have reported associations between high-quality diet with lower depression, pain, fatigue, disability and higher QoL (11, 23, 24). Prospectively, high-quality diet was associated with lower disability at 2.5-year follow-up (25) and lower depression at 10-years (26). MS-diet adherence has been positively associated with diet quality (7). Higher DSQ scores were seen in pwMS who followed Swank and Wahls diets (1). PwMS who adhered to the OMS-diet were 3.5 times more likely to have a high-quality diet indicated by DHQ scores above the median (11).

While the MS diets adherence and high-quality diet have demonstrated potential benefits on health outcomes, robust evidence supporting long-term dietary modification is limited due to insufficient sample sizes and short durations of dietary adherence (15, 27). Moreover, ongoing adherence to diet may be challenging for some due to individual barriers of health, finance, lack of support, and personal motivation (28, 29). Furthering our previous study showing that adherence to OMS- and high-quality diets are cross-sectionally associated with lower fatigue, depression, and disability (11), we assess whether level of adherence impacts health outcomes. We compare non-adherence to partial- and ongoing-adherence, to gain insights into potential health benefits, and whether these are sustained upon cessation of diet, or whether they are beneficial with later commencement. These findings may guide pwMS and health professionals on prioritization of dietary behaviors for MS management and secondary prevention.

## 2. Materials and methods

### 2.1. Study design and participants

Data were analyzed from the health outcomes and lifestyle in a sample of pwMS (HOLISM) longitudinal observational study, the methodology of which has been previously described (30). Briefly, pwMS were recruited *via* social media platforms for pwMS from October to December 2012. Consenting participants aged ≥ 18 years, and with a self-reported clinician-diagnosis of MS were eligible (*n* = 2,466). Participants completed an online survey capturing sociodemographic, clinical, lifestyle behaviors, and health data. Participants were then surveyed at 2.5-year intervals thereafter. Adherence to MS-diet was queried at 5- (2017) and 7.5-year (2019) timepoints; thus, analyses were restricted to pwMS who completed both surveys (*n* = 671, 27%). Ethical approval was granted by the University of Melbourne Human Research Ethics Committee (ID # 1545102).

### 2.2. Demographics and clinical characteristics

Age was calculated from reported dates of birth and survey completion. Sex, country of residence, highest level of education (no formal education, primary school, secondary school, vocational school, bachelor’s degree, and postgraduate degree), employment (employed in paid work, unemployed and seeking paid employment, unemployed and not seeking paid employment, stay at home parent or carer, student, retired due to age, retired due to medical reasons or disability), and perceived relative socioeconomic status (SES) (31) were queried and re-categorized (Table 1).

**TABLE 1:** Participant sociodemographic and clinical characteristics.

	Excluded participants (0-year)	Study participants (0-year)	Study participants (at 7.5-year)
	***N* = 1,795**	***N* = 671**	***N* = 671**
	**Mean (SD)**		
Age, years (mean, SD)	45.7 (10.6)	45.7 (10.2)	53.3 (10.2)
MS duration, years (mean, SD)	8.15 (7.5)	7.88 (6.9)	15.5 (6.9)
	***n* (%)**		
**Sex**			
Male	288 (16.9%)	129 (19.2%)	129 (19.2%)
Female	1,415 (83.1%)	542 (80.8%)	542 (80.8%)
**Country of residence**			
Aus/NZ	523 (29.1%)	314 (46.8%)	391 (41.2%)
UK	300 (16.7%)	119 (17.7%)**	180 (19.0%)
US/Canada	758 (42.2%)	157 (23.4%)***	253 (26.7%)
Other	214 (11.9%)	81 (12.1%)**	124 (13.1%)
**University degree**			
No	791 (44.4%)	208 (31.1%)	178 (26.8%)
Yes	992 (55.6%)	461 (68.9%)***	486 (73.2%)
**Employment**			
Paid employment	916 (51.4%)	438 (65.3%)	361 (54.6%)
Unemployed	349 (19.6%)	104 (15.5%)***	80 (12.1%)
Retired	518 (29.1%)	129 (19.2%)***	220 (33.3%)
**BMI**			
Under/healthy	966 (54.6%)	435 (64.8%)	398 (59.3%)
Overweight	412 (23.3%)	144 (21.5%)*	155 (23.1%)
Obese	393 (22.2%)	92 (13.7%)***	118 (17.6%)
**MS type**			
Non-progressive	1,105 (63.0%)	486 (73.0%)	486 (73.0%)
Progressive	393 (22.4%)	105 (15.8%)***	105 (15.8%)
Unsure/other	255 (14.6%)	75 (11.3%)**	75 (11.3%)
**Disability (P-MSSS)**			
Normal/mild	894 (54.8%)	444 (68.0%)	445 (67.1%)
Moderate	436 (26.7%)	141 (21.6%)***	153 (23.1%)
Severe	302 (18.5%)	68 (10.4%)***	65 (9.8%)
**Fatigue (FSS > 5)**			
No	749 (49.4%)	382 (61.3%)	381 (61.1%)
Yes	766 (50.6%)	241 (38.7%)***	243 (38.9%)
**Depression (PHQ-2 > 2)^a^**			
No	1,213 (77.2%)	586 (90.0%)	553 (87.0%)
Yes	359 (22.8%)	66 (10.1%)***	83 (13.1%)
**Comorbidities^b^**			
0	964 (53.7%)	411 (61.3%)	482 (71.8%)
≥ 1	831 (46.3%)	260 (38.8%)**	189 (28.2%)

^a^PHQ-2 was used at baseline (0-year) and PHQ-9 was used at 5- and 7.5-year timepoints.

^b^Number of treated comorbidities. BMI, body mass index; MS, multiple sclerosis; na, not available; PHQ, patient health questionnaire; P-MSSS, patient-derived MS severity scores; SD, standard deviation. Percentages may not total exactly 100.0% due to rounding. Difference of variables between LTFU and study sample at baseline (*p < 0.05 **p < 0.01 ***p < 0.001) were assessed by *t*-test and log-binomial regression.

MS phenotype was re-categorized into non-progressive (benign/RRMS), progressive (SPMS/PPMS/PRMS) and unsure/other; MS duration was calculated from year of diagnosis and survey completion date. Participants’ report of ongoing symptoms due to relapse in the preceding 30 days was dichotomised to No/Yes. BMI was calculated by weight/height^2^ and classified as per World Health Organization guidelines (32); underweight and normal weight were consolidated due to small sample size in the former group. Comorbidity number was assessed by self-administered comorbidity questionnaire (SCQ) (33) and dichotomised to 0 and ≥ 1. Participant use of prescription medication for depression and fatigue was also queried (No/Yes). Participants were also queried at each timepoint whether they were experiencing ongoing symptoms due to recent relapse in the preceding 30 days.

### 2.3. MS-diet adherence

Diet adherence was queried by No/Yes response to “Do you currently follow a particular MS diet?,” with a Yes response allowing multiple selection from options of Ashton Embry Best Bet, McDougall, OMS, Paleo, Swank, and Wahls. As multi-diet selection was possible, and follow-up was 2.5 years, it is possible that an individual may be represented in more than one diet group.

Duration of adherence to each MS-diet was queried by response to “How long have you been following this diet?” with options from < 12 months and 1–20 years (1-year intervals). Stringency of adherence was queried by response to “How rigorously have you followed this diet?” assessed on a 5-point Likert scale, where 1 = not rigorously at all and 5 = very rigorously. For analysis, adherence to a MS-diet was defined as ≥ 12-month duration and stringency of ≥ 3/5, consistent with our prior study (11).

### 2.4. High-quality diet adherence

Diet quality was assessed using a modified form (34) of the DHQ (35), querying intake of fruit/vegetables, takeaway, fat, fiber, food choices, and food preparation. A summary score was calculated with a possible score range of 20–100. Higher DHQ scores indicate higher quality of diet. Total DHQ score was dichotomised at the median to differentiate high- and low-quality diet (11).

MS-diet and high-quality diet adherence was defined based on adherence at each timepoint (0 = No; 1 = Yes): non- (0–0), commenced- (0–1), ceased- (1–0), and ongoing-adherence (1–1).

### 2.5. Health outcomes

Depression was measured *via* the patient health questionnaire short version (PHQ-2) at baseline (0-year), which contains two items inquiring about the frequency of depressed mood over the past 2 weeks on a 4-point Likert scale (0 = not at all to 3 = nearly every day). The PHQ-2 score ranges from 0 to 6 and with scores > 2 indicate major depressive disorder (36). At 5- and 7.5-year timepoints, depression was measured *via* the PHQ-9. The PHQ-9 includes PHQ-2 and additional 7 items on depression; total scores range from 0 to 27 and score > 4 represent depressive symptoms (37).

Fatigue was measured by the 9-item fatigue severity scale (FSS), where a mean score > 5 was defined as clinically significant fatigue (38).

Disability status was measured by the patient determined disease steps [PDDS; (39)], from which the disease duration-adjusted patient-determined MS severity score (P-MSSS) was derived and categorized as normal/mild (0–3), moderate (4–5) and severe (6–8) disability (40).

### 2.6. Statistical analysis

All analyses were conducted in Stata version 16.0 (StataCorp, College Park, USA). Differences in cohort characteristics between the analysis sample and those lost to follow-up (LTFU) were assessed by *t*-test and log-binomial regression for continuous and binary/categorical variables, respectively. Statistical significance was set at *p* < 0.05.

Only adherence to OMS-diet was analyzed individually, as other MS diets had too few participants adhering. Participants followed OMS-diet was tested against not following OMS-diet. Associations between OMS-diet and high-quality diet (DHQ scores > median) adherence at 5- to 7.5-year with depression and fatigue were assessed by log-binomial regression models. Categorical disability was compared between normal/mild vs. moderate, normal/mild vs. severe and moderate vs. severe, using log-binomial regression models. Risk ratios (RR) and 95% confidence intervals (CI) were generated. All models were adjusted for ongoing symptoms from recent relapse at 5- and 7.5-years, and clinical outcomes at 5-years. Models were further adjusted for age, sex, perceived SES, education, employment, MS duration, disability (for fatigue and depression), fatigue (for disability), number of comorbidities, and use of antidepressant and anti-fatigue medication (for fatigue and depression).

## 3. Results

### 3.1. Participant characteristics

Of 2,466 baseline HOLISM participants, 671 (27%) completed 5- and 7.5-year surveys and were included in the study (Table 1). Compared to participants LTFU (73%), the included participants were more likely to be residents of Australia or New Zealand, university educated, employed, of non-progressive MS type, having normal/mild disability, and less likely to be overweight or obese, or to have fatigue, depression, or ≥ 1 comorbidity.

Study participants in the analysis sample at 7.5-year timepoint were predominantly female, of mean age 53 years, 41% living in Australia or New Zealand, 73% with a university degree, and 55% undertaking paid employment. A majority were of non-progressive MS type, and mean MS duration was 16 years. Most participants were of underweight/healthy BMI, 67% of participants had normal/mild disability, 58% reported fatigue, 51% reported depression, and 28% reported ≥ 1 comorbidity.

### 3.2. Adherence to MS-diet and high-quality diet

Overall, 54% of pwMS reported adherence to a MS-diet at 5-years; comprising 44% OMS, 7% Swank, and 7% other diets (Table 2). Ongoing-adherence was higher than ceased or commenced in most MS diet programs. The highest rate of ongoing-adherence was for OMS-diet, with 76% (221/292) adhering at 5- and 7.5-years and 11% (71/292) ceasing. Of the 56% (379/671) of pwMS who did not follow OMS-diet at 5-years, 5% (34/379) commenced-adherence at 7.5-years.

**TABLE 2:** MS-diet adherence from 5- to 7.5-year (*n* = 671, 100%).

Adherence at 5-year	No (0)			Yes (1)		
**Adherence at 7.5-year**	**Non (0–0)**	**Commenced (0–1)**	**Total**	**Ceased (1–0)**	**Persistent (1–1)**	**Total**
MS-diet^a^	269 (40%)	41 (6%)	310 (46%)	81 (12%)	280 (42%)	361 (54%)
OMS	345 (51%)	34 (5%)	379 (56%)	71 (11%)	221 (33%)	292 (44%)
Swank	613 (91%)	13 (2%)	626 (93%)	20 (3%)	25 (4%)	45 (7%)
Wahls	648 (97%)	5 (1%)	653 (97%)	8 (1%)	10 (1%)	18 (3%)
Paleo	652 (97%)	7 (1%)	659 (98%)	8 (1%)	4 (1%)	12 (2%)
Ashton Embry Best Bet	665 (99%)	1 (0%)	666 (99%)	1 (0%)	4 (1%)	5 (1%)
McDougall	664 (99%)	4 (1%)	668 (99%)	2 (1%)	1 (0%)	3 (1%)
High-quality diet^b^	267 (43%)	47 (8%)	314 (51%)	47 (8%)	255 (41%)	302 (49%)

^a^Participants adhered to any of the six MS-diet. Total of MS-diet is less than the total of individual diets as some participants selected multiple diets.

^b^DHQ score above the medium. Percentages based on total *n* = 671 participants (100%); percentages may not total 100 due to rounding.

Ongoing-adherence to Swank-diet was lower: 56% (25/45) adhered at both timepoints, 44% ceased. Of 93% (626/671) pwMS who did not adhere to Swank-diet at 5-year, only 2% commenced-adherence at 7.5-years. Very few pwMS adhered to other MS diets, ranging from 3% (Wahls diet) to below 1% (McDougall diet) at 5-years.

Ongoing-adherence to high-quality diet was 41% (Table 2); 8% of pwMS increased (commenced) or decreased (ceased) diet quality from 5- to 7.5-years.

### 3.3. Associations between OMS-diet adherence and health outcomes

**FIGURE 1:**
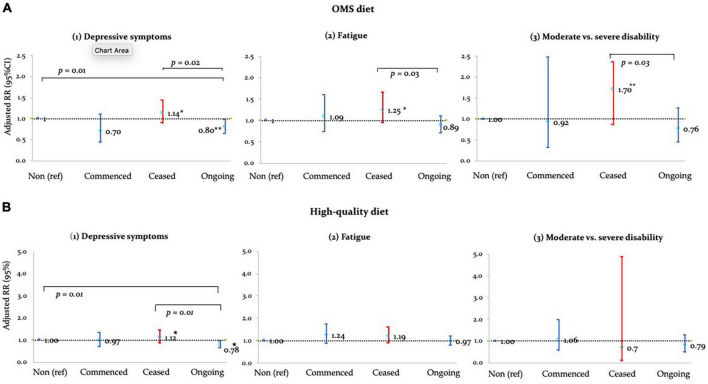
Adjusted RRs of **(A)** OMS- and **(B)** high-quality diet adherence vs. health outcomes. *p < 0.05; **p < 0.01.

Ongoing-adherence to OMS-diet was associated with lower relative risk of depressive symptoms, fatigue and disability compared to non- and/or ceased-adherence to the diet (Table 3 and Figure 1A). Ongoing-adherence to OMS-diet had 20% (RR = 0.80, *p* < 0.05) lower relative risk of depressive symptoms than non-adherence and 30% (RR = 0.70, *p* < 0.01) lower relative risk of depressive symptoms than ceased-adherence. Ongoing-adherence to OMS-diet also had 29% (RR = 0.71, *p* < 0.05) lower relative risk of fatigue compared to ceased-adherence (Figure 1A). For disability, ongoing-adherence to OMS-diet had 57% (RR = 0.43, *p* < 0.05) lower relative risk of severe rather than moderate disability compared to ceased-adherence (Figure 1A). Significant differences were not observed between non-adherence and ceased/commenced-adherence, or between commenced- and ongoing-adherence.

**TABLE 3:** Associations between OMS-diet and high-quality diet adherence with 7.5 years health outcomes (*N* = 671).

	Depression	Fatigue	Disability		
**Diet adherence**	**PHQ > 4**	**FSS > 5**	**Normal/mild vs. moderate**	**Normal/mild vs. severe**	**Moderate vs. severe**
	**aRR (95% CI)**				
**OMS diet^a^**					
None	1.00 [Ref]	1.00 [Ref]	1.00 [Ref]	1.00 [Ref]	1.00 [Ref]
Commenced	0.70 (0.44, 1.11)	1.09 (0.74, 1.60)	1.03 (0.57, 1.85)	2.07 (0.75, 5.69)	0.92 (0.32, 2.67)
Ceased	1.14 (0.90, 1.44)	1.25 (0.95, 1.66)	0.95 (0.59, 1.52)	1.51 (0.82, 2.75)	1.70 (0.86, 3.35)
Ongoing	**0.80 (0.65, 0.97)** **	0.89 (0.71, 1.11)	1.11 (0.84, 1.48)	0.83 (0.58, 1.19)	0.76 (0.45, 1.26)
	**aRR (95% CI)**				
**High-quality diet^b^**					
None	1.00 [Ref]	1.00 [Ref]	1.00 [Ref]	1.00 [Ref]	1.00 [Ref]
Commenced	0.97 (0.70, 1.33)	1.24 (0.88, 1.74)	1.07 (0.65, 1.74)	1.22 (0.64, 2.31)	1.06 (0.57, 1.98)
Ceased	1.12 (0.87, 1.44)	1.19 (0.89, 1.59)	0.87 (0.54, 1.41)	1.22 (0.32, 4.60)	0.70 (0.10, 4.88)
Ongoing	**0.78 (0.64, 0.94)** **	0.97 (0.79, 1.20)	1.19 (0.91, 1.55)	1.14 (0.71, 1.84)	0.79 (0.49, 1.28)

^a^OMS-diet is compared against not following OMS-diet.

^b^High-quality diet is compared against DHQ scores below median. Models adjusted for 5- and 7.5-year ongoing symptoms due to recent relapse, 5-year outcomes, age, sex, MS phenotype, MS duration, perceived SES, education, employment, number of treated comorbidities, disability (for fatigue and depression), and use of antidepressant and anti-fatigue medication. Boldface denotes statistical significance (**p < 0.01). aRR, adjusted risk ratio; DHQ, diet habits questionnaire; FSS, fatigue severity scale; OMS, Overcoming MS.

### 3.4. Associations between high-quality diet and health outcomes

Adherence to high-quality diet was associated with depressive symptoms, but not fatigue and disability (Table 3 and Figure 1B). Compared to non-adherence, ongoing-adherence to high-quality diet was associated with 22% (RR = 0.78, *p* < 0.05) lower relative risk of depressive symptoms (Table 3). Ongoing-adherence was also associated with 30% (RR = 0.70, *p* < 0.05) lower relative risk of depressive symptoms than ceased-, but not commenced-, adherence (Figure 1B).

## 4. Discussion

While diet has been associated with positive health outcomes in pwMS, the role of ongoing diet-adherence is under-explored. We compared non-adherence to the OMS- and high-quality diet to partial and ongoing-adherence at two timepoints over 2.5-year period on depressive symptoms, fatigue, and disability. Compared to non- or ceased-, ongoing-adherence was associated with optimal health outcomes in all analyses. Ongoing-adherence to the OMS- or a high-quality diet was associated with lower depressive symptoms than non- and ceased-adherence; and ongoing-adherence to the OMS-diet was also associated with lower fatigue and severe disability than ceased-adherence. No difference in health outcomes was observed between commenced and other adherence levels.

The study population was 81% female, 73% reporting non-progressive MS type, and the majority with mild disability as well as university educated, as reported in other MS cohorts (23, 24). The analysis population comprised 27% of baseline participants, with characteristics of less severe disability, higher education, more Australian and New Zealand residents, employed, and were less likely to have progressive MS type, have one or more comorbidities, or to report fatigue or depression, compared to excluded participants. The generalisability of these results may thus be limited. A range of demographics and clinical confounders were included in statistical models to adjust for these and other potential biases.

Fifty percent of the analysis reported having adhered to a MS-diet for at least 12-month at the 5-year timepoint: 44% to OMS, 7% to Swank, 1.5% to Wahls, and < 1.5% to other MS-diet. The proportions are similar to prior studies, such as a USA longitudinal study of 6,990 pwMS that reported although 45% of participants modified their diet after their MS diagnosis, only 2% followed a MS-diet specifically Swank or Wahls (1). A survey of 337 pwMS reported 42% adhering a MS-diet in Germany (41) and 11% of 428 pwMS in South Australia followed the Swank-diet (42). The proportion of pwMS adhering to the OMS-diet in the current study is markedly higher than the 6–20% reported in previous studies (24, 43), possibly reflective of recruitment primarily through sites promoting healthy lifestyle behaviors (30), as well as participants’ awareness of and engagement with the multimodal OMS lifestyle program (44).

In the current study, in addition to diet-adherence, we assessed ongoing-adherence at 5- and 7.5-year timepoints, which was high for all diets: 75% for OMS, 56% for Swank, and 61% for other diets, suggestive of commitment to dietary modification by pwMS. Previous studies have shown 75–90% pwMS adhered to a Mediterranean diet at 6-month follow-up (20, 45) and 50% of pwMS followed Swank-diet rigorously for 34 years (4). While diet commitment for an extended period can be challenging, our data show that it is achievable by pwMS.

Ongoing-adherence to OMS-diet was associated with 20–30% lower risk of depressive symptoms than both non- and ceased-adherence. These results corresponding with our cross-sectional study showing 27% lower depression associated with adherence to OMS-diet at 5-years (11). The OMS-diet recommends a low saturated-fat, plant-based whole food diet plus seafood; this diet has been shown to be a high-quality diet (23, 24), and adherence to OMS-diet has been found to be associated with 10-point higher DHQ scores (7). While the mechanisms linking MS-diet to depression are uncertain, a role of the gut-brain axis has been suggested (27). Inflammation is known to play a key role in MS progression (46) and the anti-inflammatory and neuroprotective effects of fruit/vegetables and of low saturated fat diet have been documented (9, 47, 48). Our findings may be in part explained by the anti-inflammatory effects of a low saturated fat diet such as the OMS-diet. The benefits of OMS-diet on depressive symptoms were not observed in pwMS who commenced of the diet at 7.5-year, suggesting that early and ongoing-adherence may be required for reduced depressive symptoms.

Ongoing-adherence to OMS-diet was associated with improved fatigue or disability compared to ceased-, but not compared to non- or commenced-adherence. These results suggest diet is unlikely the only factor that contributes to better outcomes, a multimodal lifestyle approach may be best. Compared to ceased- ongoing-adherence was associated with 29% lower fatigue and 26% lower severe disability, suggesting benefits are not sustained if OMS-diet adherence ceases. Prior studies have also shown high-quality diet associated with 30 and 44% lower fatigue and disability, respectively (11). Diet modification may affect fatigue *via* modulation of inflammation or oxidative stress (9, 13, 14); therefore, anti-inflammatory diets could be a potential intervention for pwMS. However, ongoing-adherence did not show better outcomes in fatigue and disability than non- and commenced-adherence, results should therefore be interpreted with caution. While there are potential benefits of ongoing-adherence to OMS-diet on fatigue and disability, possibility for reverse causality in which people ceased-adherence due to those symptoms exists, warrants further study. MS is a chronic disease requires long-term management. Fatigue and disability have been reported by pwMS as common barriers for lifestyle modification (28). Current results show a robust beneficial impact of ongoing-adherence with depressive symptoms may suggest that pwMS are more able to adhere dietary modification despite those symptoms, while fatigue and disability are stronger barriers for sustained engagement. However, conclusions could not be fully drawn with the current data and future longitudinal assessments are required to ascertain the associations. Regardless of potential reverse causality, support to improve diet-adherence, especially for pwMS experiencing fatigue and disability symptoms, are important.

Ongoing-adherence to high-quality diet at both timepoints over a 2.5-year period, compared to non- and ceased-adherence, had 22% and 33% lower depressive symptoms, respectively. This aligns with reported observations of pwMS who maintained a high-quality diet over 11-years had fewer symptoms of depression compared to those whose diet quality was consistently low or worsened over time (49). Current results are also concordant with studies showing high-quality diet cross-sectionally associated with lower depression (23, 24). Together the results support ongoing-adherence to a high-quality diet may improve depression in pwMS. No difference was observed between high-quality diet adherence and fatigue or disability, partially contradictory to our prior prospective findings from 0 to 2.5-years that showed 36–41% lower risk of disability progression but no association with fatigue (24). The disparity may be due to less generalizable population in the current study, showing healthy participant bias including < 10% prevalence of disability, lower than the reported 14% in pwMS (25), and more likely to be adhering to multimodal healthy lifestyle behaviors (25). Both ongoing-adherence to OMS- and high-quality diet showed benefits on depressive symptoms, suggesting that sustained diet that is high in fruits, vegetables, whole grains, and fish, and low in saturated fat, refined sugar and processed meat should be encouraged. However, only ongoing-adherence to OMS-diet showed reduced fatigue and disability than ceased-adherence. This suggests important elements of the OMS-diet for MS management such as low saturated fat and omega-3 supplementation. Alternatively, it may be that pwMS with fatigue and disability are more likely to cease a restrictive MS-diet. Future longitudinal studies are needed to determine the associations.

No differences in associations with health outcomes were observed between non- and commenced-adherence to either OMS- or high-quality diet, nor between commenced- and ceased- or ongoing-adherence. These may suggest that early dietary modification is needed to observe the benefits, however, it is possible that no association was found due to small size in the commenced-adherence group, the subjective measure of diet adherence in our study, as well as our study population adhering to other healthy lifestyle behaviors that are also associated with improved health outcomes (50, 51). Future research assessing individual and additive impacts of lifestyle behaviors, as well as adherence and duration information may provide further insight.

A limitation of our study was 73% attrition of baseline HOLISM participants; while characteristics of the analysis population were comparable with other MS cohorts, they may not be representative of the broader population of pwMS in the real world, and thus our results need to be interpreted with caution. Healthy participant bias is acknowledged; and compared to participants LTFU, pwMS returning at 5-year timepoint, had adopted healthy behaviors and engaged with information on a multimodal lifestyle program for pwMS (44). Therefore, associations between OMS-diet and health outcomes may be due to adoption of multiple healthy lifestyle behaviors. Future studies may consider assessing associations of individual lifestyle behaviors independently and potential additive effects. There is the potential for participants to select more than one diet, and non-specific querying of foods and drinks that were adhered to, as well as self-assessment of degree of adherence based on Likert scale, limits data accuracy. Additionally, whether diet was adhered to for the entire 2.5-year interval was not assessed. It is possible that pwMS may have altered their diet type and/or stringency of adherence in between the two timepoints. Future studies may consider assessing diet and level of adherence using validated tools that allow substantiation of self-defined labels of adherence. Few pwMS commenced-adherence from 5- to 7.5-year timepoints, which may account for insignificant findings in this group. Finally, the number of pwMS adhering to MS-diets other than OMS was few, therefore associations for other MS-diets on health outcomes should be investigated in larger populations.

Nonetheless, the study is the first to our knowledge to assess associations between partial- and ongoing-adherence on health outcomes over a 2.5-year period. The strengths of our study include that the analysis comprised 671 participants from 33 countries and therefore having exposure to different diet guidelines for MS management. The survey includes a comprehensive collection of demographic and clinical characteristics, enabling appropriate adjustment for selection bias and relevant confounders. Additionally, by restricting adherence to being ≥12 months and ≥3/5 rigour, we were able to ensure true adherence rather than brief for diet modification.

## 5. Conclusion

Adherence to a MS- or high-quality diet over 2.5-year was high, suggesting ongoing-adherence to diet is acceptable and achievable by pwMS. Ongoing-adherence to the OMS-diet may help improve MS outcomes, especially depressive symptoms; however, further assessments are required to confirm the causality. Partial-adherence was not associated with better outcomes than non-adherence. These findings suggest that potential benefits of diet require ongoing efforts, therefore care management should consider methods to support pwMS to maintain high-quality diet. Ongoing-adherence to MS-diet may be more challenging for pwMS with fatigue and disability. Healthcare providers should consider strategies and tools that are tailored to the individual’s needs. Future studies assessing ongoing-adherence to other MS-diets would be worthwhile.

## Data availability statement

The raw data may not be shared due to conditions approved by our institutional ethics committee. Access to de-identified aggregate group data may be requested through SN or NN, nnag@unimelb.edu.au.

## Ethics statement

The studies involving human participants were reviewed and approved by the University of Melbourne Human Research Ethics Committee (ID# 1545102). The patients/participants provided their written informed consent to participate in this study.

## Author contributions

NN: conceptualization, visualization, supervision, and project administration. SS-Y and MY: data curation. NN and MY: methodology and writing—original draft preparation. MY: formal analysis. GJ and SN: resources. NN, MY, SS-Y, GJ, and SN: writing—review and editing. GJ: funding acquisition. All authors approved the final version of the manuscript.
